# Computed Tomography Image Processing Analysis in COVID-19 Patient Follow-Up Assessment

**DOI:** 10.1155/2021/8869372

**Published:** 2021-04-29

**Authors:** Santiago Tello-Mijares, Luisa Woo

**Affiliations:** ^1^Postgraduate Department, Instituto Tecnológico Superior de Lerdo, 35150 Lerdo DGO, Mexico; ^2^Medical Familiar Unit, Instituto de Seguridad y Servicios Sociales de Los Trabajadores del Estado, 27268 Torreón COAH, Mexico

## Abstract

The rapid worldwide spread of the COVID-19 pandemic has infected patients around the world in a short space of time. Chest computed tomography (CT) images of patients who are infected with COVID-19 can offer early diagnosis and efficient forecast monitoring at a low cost. The diagnosis of COVID-19 on CT in an automated way can speed up many tasks and the application of medical treatments. This can help complement reverse transcription-polymerase chain reaction (RT-PCR) diagnosis. The aim of this work is to develop a system that automatically identifies ground-glass opacity (GGO) and pulmonary infiltrates (PIs) on CT images from patients with COVID-19. The purpose is to assess the disease progression during the patient's follow-up assessment and evaluation. We propose an efficient methodology that incorporates oversegmentation mean shift followed by superpixel-SLIC (simple linear iterative clustering) algorithm on CT images with COVID-19 for pulmonary parenchyma segmentation. To identify the pulmonary parenchyma, we described each superpixel cluster according to its position, grey intensity, second-order texture, and spatial-context-saliency features to classify by a tree random forest (TRF). Second, by applying the watershed segmentation to the mean-shift clusters, only pulmonary parenchyma segmentation-identified zones showed GGO and PI based on the description of each watershed cluster of its position, grey intensity, gradient entropy, second-order texture, Euclidean position to the border region of the PI zone, and global saliency features, after using TRF. Our classification results for pulmonary parenchyma identification on CT images with COVID-19 had a precision of over 92% and recall of over 92% on twofold cross validation. For GGO, the PI identification showed 96% precision and 96% recall on twofold cross validation.

## 1. Introduction

The COVID-19 pandemic is an infectious disease that has affected millions of individuals all over the world, and it has caused thousands of deaths since December 19, 2019, according to the World Health Organization (WHO) [1]. On January 30, 2020, the WHO designated the outbreak of this novel coronavirus that had not been seen before in humans to be a “public health emergency of international concern.” This was followed by the declaration of a pandemic on March 11, 2020 [1, 2]. COVID-19 presents a significant challenge to medical professions due to the widespread effect of this pandemic. Its influence on the practice of diagnosis and monitoring of ground-glass opacity (GGO) and pulmonary infiltrate (PI) by medical image processing is the subject of this work.

GGO is a descriptive term referring to an area of increased attenuation in the lung on computed tomography (CT) with preserved bronchial and vascular markings. It is a nonspecific sign with a wide etiology including infection, chronic interstitial disease, and acute alveolar disease. PI is a substance that is denser than air, such as pus, blood, or protein, which lingers within the lung parenchyma. PI is associated with pneumonia, tuberculosis, nocardiosis, and now COVID-19. PI can be observed on a CT.

CT can help complement a reverse transcription-polymerase chain reaction (RT-PCR) diagnosis. CT was also demonstrated to be effective in the current diagnosis, including follow-up assessment, and in the evaluation of disease evolution [3, 4]. Different clinical studies have shown that chest CT imaging can be helpful in supporting early detection of COVID-19 [5–7]. Latif et al. [8] present a comprehensive review and they attempt to systematize ongoing data science activities in this area.

Since the COVID-19 outbreak was identified in December 2019, there have been increasing efforts to develop different artificial intelligence methods to screen patients for COVID-19 based on medical images such as CT scans [9–26].

Wang et al. [9] used deep-learning methods to detect radiographical changes in COVID-19 patients. Chen et al. [10] proposed the UNet++ architecture to detect suspicious lesions on CT scans. Ophir et al. [11] used two- and three-dimensional convolutional neural networks (CNNs) to calculate the corona score (which represents the evolution of the COVID-19 infection in the lungs).

Li et al. [12] developed a neural network (COVNet) to extract visual features and modified a residual network with 50 layers to detect COVID-19 using CT scans. Mobiny et al. [13] developed a Detail-Oriented Capsule Networks (DECAPS) learning architecture that could identify fine-grained and distinguishing image features to classify COVID-19 based on CT scans. Wang et al. [14] introduced a COVID-Net for the automatic interpretation of chest radiographs from COVID patients.

Chaganti et al. [15] proposed a system that identifies suspicious lung regions in CT scans using a deep reinforcement learning algorithm, and they measured the abnormality and severity caused by COVID-19. Butt et al. [16] extracted features from CT scans using an architecture based on ResNet18, to identify segments of pathological lung regions that then serve as the input to a classifier for predicting COVID-19 disease. Al-Karawi et al. [17] showed the development and performance of machine learning schemes by an innovative frequency domain algorithm, which is called an FFT-Gabor scheme. He et al. [18] proposed a Self-Trans approach, which synergistically integrates contrastive self-supervised learning with transfer learning to learn powerful and unbiased feature representations for reducing the risk of overfitting.

Chowdhury et al. used CNN to identify COVID-19 patients based on chest X-ray images [19]. Yang et al. developed a deep-learning-based CT diagnosis system (DeepPneumonia) to assist clinicians with identifying patients with COVID-19 [20]. Shan et al. used the “VB-Net” neural network to segment COVID-19 infection regions in CT scans [21]. Shen et al. proposed an infection-size-aware random forest (iSARF) method that can automatically categorize subjects into groups with different ranges of infected lesion sizes [22]. Polsinelli et al. [23] proposed a light CNN design based on the SqueezeNet model, as an efficient way to distinguish between COVID-19 CT images and other CT images (community-acquired pneumonia and/or healthy images).

Fan et al. [24] proposed a novel COVID-19 lung infection segmentation deep network (Inf-Net) to automatically identify infected regions from the chest CT scans. Amyar et al. [25] proposed a multitask deep-learning model to jointly identify COVID-19 patients and distinguish COVID-19 lesions from chest CT images. Wu et al. [26] developed a novel joint classification and segmentation (JCS) system to allow a real-time and explainable COVID-19 diagnosis.

**Figure 1:**
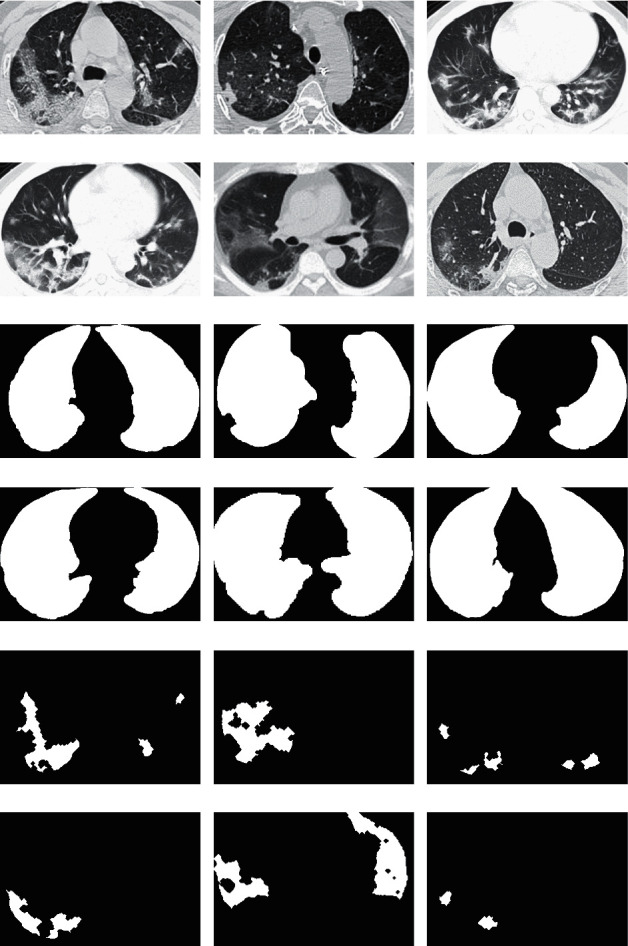
Input images and ground truth. (a–f) Computed tomography (CT) in a patient with COVID-19, (g–l) ground-truth data for identification of pulmonary parenchyma, and (m–r) ground-truth data for ground-glass opacity (GGO) and pulmonary infiltrate (PI) identification.

Here, we propose an effective and efficient method for segmenting and identifying GGO and PI in patients with typical COVID-19 CT images from the Zhao dataset [27] (Figure 1(a)–1(f)). Additionally, the ground truth was preset for pulmonary parenchyma (Figure 1(g)–1(l)), GGO, and PI (Figure 1(m) and 1(n)). We showed excellent results based on precision and recall using a set of real, practical, complex, and representative data.

**Figure 2:**
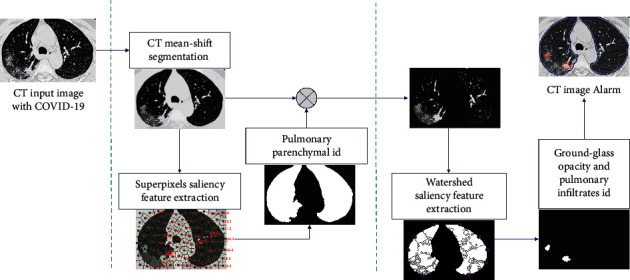
Overall method description.

Our motivation is to contribute a robust two-level classifier system to help improve both the segmentation and classification of pulmonary parenchyma from CT scans of patients with COVID-19 based on GGO and PI identification and then have the medical expert perform the patient assessment and evaluation at a later time. Our work is a medical aid tool. The next section of this paper describes the proposed segmentation and the two-level cascade classification algorithm. The segmentation method was based on a mean shift for oversegmentation followed by morphologic and superpixel-SLIC clustering process for regrouping the pulmonary parenchyma CT images using a cluster feature extraction process and tree random forest (TRF) as the first objective. Next, watershed segmentation was applied to the mean-shift clusters only in the identified pulmonary parenchyma zone, followed by identification of the GGO and PI using the watershed cluster feature extraction. They were then classified using the TRF method.  Section 3 presents the classification results that were obtained, and Section 4 discusses the results and concludes the paper.  Figure 2 illustrates the stages of the proposed method.

## 2. Materials and Methods

### 2.1. Dataset

The dataset was provided by Zhao (2020) [27] (Figure 1; images from the Zhao (2020) dataset [27]). They built a publicly available COVID-CT dataset that contained 349 CT images from patients who were positive for COVID-19. The images were not captured under the same conditions, and therefore, the resolution was different and the images were preprocessed. In this study, MATLAB was used to process all of the images, such as performing image preprocessing, segmentation, and feature extraction. This is discussed in detail in the sections below.

### 2.2. Image Oversegmentation

**Figure 3:**
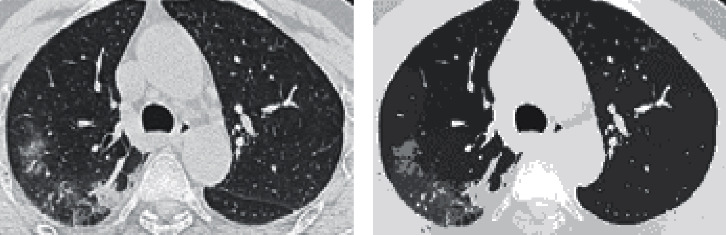
Image oversegmentation. (a) Input grey-scale image, (b) Mean-shift L band image clusters.

Input grey images were first truncated by setting the two least significant bits of every pixel and channel to 0, thereby obtaining images with 64 levels. These images were coded as 0, 4, 8,…, 252 for simplicity. This step assumes normalization of the CT input images, and it removed noise and perceptually irrelevant details. It also resized all the 200×300 pixels (Figure 3(a)).

This first segmentation stage consisted of oversegmentation. We propose the use of the mean-shift algorithm [28] to obtain an oversegmented image. Mean shift is a nonparametric technique for analyzing multimodal data. It has multiple applications in pattern analysis [29] and image segmentation. We started from the observation that regions that belonged to the pulmonary parenchyma should be near the center and connected, and they should all be characterized by similar intensity and texture values; additionally, they are always much darker than the surrounding nonchest regions (body and background). We characterized each image pixel using a vector [*L,x,y*], where [*L*] corresponds to its intensity and [*x,y*] corresponds to its coordinates. We then can use the mean-shift algorithm over this three-dimensional distribution with a band-width value *h* *=* *20*, which was selected so that the chest was segmented into more than one region; this is required for the later regrouping process to be effective.

One goal of this paper is to develop an automatic and robust mean-shift and superpixel-SLIC method for accurate segmentation of pulmonary parenchyma identification in CT imaging.

**Figure 4:**
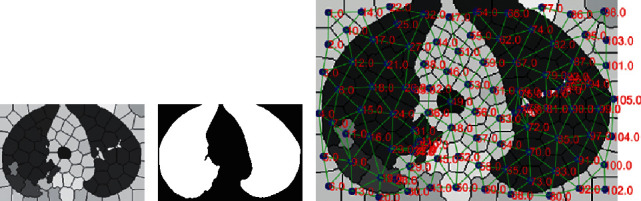
Pulmonary parenchyma identification. (a) Superpixel-SLIC segmentation, (b) result from tree random forests (TRF), (c) and spatial-context-saliency feature extraction and connections.

The segmented image (Figure 3(b)) contains a set of clusters that are each characterized by a vector of [*L*] values. To finally identify a single region in a candidate pulmonary parenchyma, we proposed an effective regrouping technique based on superpixel-SLIC approaches (see below, Figure 4(a)).

One of the original contributions of this paper is that our first segmentation stage is built on the mean-shift and superpixel-SLIC combination. In this paper, a superpixel-SLIC is a connected set of pixels that share similar properties. We obtain superpixel-SLIC using three-dimensional mean-shift clustering that incorporates joint spatial and intensity features. The superpixel-SLIC expresses the local structure of the data in the three-dimensional feature space. Mean-shift clustering reduces data variation, thereby helping to produce accurate segmentation. By connecting superpixel-SLIC instead of pixels, the algorithm produces better results.

### 2.3. Pulmonary Parenchyma Identification

The first objective was to regroup the pulmonary parenchyma (Figure 4(b)), the body, and the background. We used superpixel-SLIC approaches over the mean-shift L band to regroup the oversegmented image clusters (Figure 3(b)), and we used the simple linear iterative clustering (SLIC) algorithm [30]. This algorithm groups pixels into regions with similar values based on their intensity similarity and proximity in the image joint spatial. Region size and high compactness, which are the two parameters of SLIC, were 100 and 20, respectively. Twenty-four iterations were used in the clustering phase of the algorithm. The next step was to obtain the average value of each new object in the superpixel-SLIC L image (Figure 4(a)) using the oversegmented image. Applying morphology and superpixel-SLIC based on the oversegmented mean-shift clustered image has some advantages for regrouping (Figure 4(a)).

From the new image, which contained the grouped pulmonary parenchyma parts and the body and the background parts (Figure 4(a)), we extracted the characteristics of the position, grey intensity, texture, and mainly the spatial-context-saliency to classify pulmonary parenchyma and nonpulmonary parenchyma (body and the background, Figure 4(b)). In Figure 4(c), 105 objects (numbers in red) and 276 connections between clusters (green lines) are shown. The blue dots are the Cartesian coordinates [x, y] of each superpixel-SLIC centroid, the pulmonary parenchyma is identified, and the spatial-context-saliency is shown by the green lines (see below).

We obtained a grey intensity feature, and one way to distinguish between different textures is to compare L levels using first-order statistics. First-order statistics were calculated based on the probability of observing a particular pixel value at a randomly chosen location in the image. They depend only on individual pixel values and not on the interaction of neighboring pixel values. The average (1) is the mean of the sum of all of the intensity values in the image. This was selected because of the specific intensity appearance of the chest, including average pixel values in L grey intensity space (*µ*_*SPc*_) of the aforementioned clusters that were obtained from the statistical average of the superpixel-SLIC clusters image (Figure 4(a)).

(1) $$\begin{array}{c} \mu_{SPc}=\frac{1}{ij}\sum_{i,j}p\left(i,j\right). \end{array}$$

We extracted the following four second-order statistics texture features: (1) contrast; (2) correlation; (3) energy; and (4) local homogeneity from the grey-level cooccurrence matrix (GLCM), which is the matrix *M*, as shown below (the measures are calculated for *d* = 0°). Haralick's GLCM [31] has been used successfully for biomedical image classification [32, 33]. We used the cooccurrence matrix for the entire L intensity. The contrast descriptor (*cn*_*SPc*_) is a measure of local variation in the image. It has a high value when the region within the range of the window has high contrast. Correlation (*cr*_*SPc*_) of the texture measures the relationship between the different intensities of grey. Mathematically, the correlation increases when the variance is low, meaning that the matrix elements are not far from the main diagonal. Energy (*e*_*SPc*_) is the sum of the squared elements in the matrix the grey-level cooccurrence, which is also known as the uniformity or the second element of angular momentum. Local homogeneity (*LH*_*SPc*_) provides information about the local regularity of the texture. When the elements of the cooccurrence matrix are closer to the main diagonal, the value of the local homogeneity is higher.

(2) $$\begin{array}{c} cn_{SPc}=\sum_{i,j}{\left|i-j\right|}^{2}M\left(i,j\right), \end{array}$$

(3) $$\begin{array}{c} cr_{SPc}=\sum_{i,j}\frac{\left(i-\mu i\right)\left(j-\mu j\right)\vec{p}\left(i,j\right)}{\sigma_{i}\sigma_{j}}, \end{array}$$

(4) $$\begin{array}{c} e_{SPc}=\sum_{i,j}p\left(i,j\right), \end{array}$$

(5) $$\begin{array}{c} LH_{SPc}=\sum_{i,j}\frac{p\left(i,j\right)}{1-\left|i-j\right|}. \end{array}$$

In equation (3), *μ* and *σ* are the mean and standard deviation values of GLCM in each row (or column, because of symmetry), respectively. We used the original quantized grey image to analyze the original texture in one direction (*d* = 0° radians), which was used to construct the GLCM.

Twelve spatial-context-saliency features, which were selected to obtain the characteristics of each object with respect to its neighbors, were based on the salience of each object obtained in a global way (i.e., the comparison of each object with respect to the other objects in the image) [34]. These features use element uniqueness *Ui* (intensity or texture) and element distribution *Di* (position).

Based on Perazzi et al. [34], we obtained spatial-context-saliency features in a local way (i.e., by comparing each object with respect to its neighbors in the image) using the objects that were grouped by the superpixel-SLIC (Figure 4(a)). We propose a four-element uniqueness *Ui* (using grey intensity and histograms). Each object *i* has a different set of *j* to *m* neighbors; *ci* and *cj* *=* *{c1, c2, ... cm}* represent the value of the intensity (average *µ*_*SPc*_) and also represent the value of the texture (cn_*SPc*_, cr_*SPc*_, e_*SPc*_, and HL_*SPc*_ of the grey band L original); and *hi* and *hj* *=* *{h1, h2, ... hm}* are the histograms of 64 bins that are obtained from the original quantized image. Furthermore, the values pi and *pj* *=* *{p1, p2, ... pm}* represent the values of the position [*x, y*] of each object.

The 105 objects and 276 connections (shown in Figure 4(c)) use the histogram values, and we obtained the Euclidean distance ED (6) and the Bhattacharyya distance BD (7) of each object *hi* with respect to its neighbor *hj*. Additionally, with the grey (average *μ*_*SPc*_) and the texture values (cn_*SPc*_, cr_*SPc*_, e_*SPc*_, and HL_*SPc*_), we obtained the average of the difference (8) and the energy (9) of each object *ci* with respect to its neighbors *cj*.

We used the Gaussian weight of the position *w*_*ij*_^*(p)*^ (10), and we effectively combined the local contrast estimation with the control of the radius of influence for the singularity operator. The local function *w*_*ij*_^*(p)*^ yields a local contrast term, which tends to overemphasize object boundaries in the saliency estimation, as seen in [34–36]. Additionally, *σ*_*p*_ controls the range of the uniqueness operator and is the variance of the distances of the position *p*_*i*_ for object *i* to positions *p*_*j*_ for object *j*.

(6) $$\begin{array}{c} ED_{i}=\frac{\sum_{j=1}^{m}\left(\sum_{x\in X}\sqrt{{\left(h_{i}\left(x\right)-h_{j}\left(x\right)\right)}^{2}}\right)w_{i,j}^{\left(p\right)}}{m}, \end{array}$$

(7) $$\begin{array}{c} BD_{i}=\frac{\sum_{j=1}^{m}\left(-\ln\sum_{x\in X}\sqrt{h_{i}\left(x\right)h_{j}\left(x\right)}\right)w_{i,j}^{\left(p\right)}}{m}, \end{array}$$

(8) $$\begin{array}{c} d_{i}=\frac{\sum_{j=1}^{m}\left(c_{i}-c_{j}\right)w_{i,j}^{\left(p\right)}}{m}, \end{array}$$

(9) $$\begin{array}{c} e_{i}=\frac{\sum_{j=1}^{m}\left|c_{i}-c_{j}\right|w_{i,j}^{\left(p\right)}}{m}, \end{array}$$

(10) $$\begin{array}{c} w_{i,j}^{\left(p\right)}=\frac{1}{\sqrt{2\pi \sigma_{p}^{2}}}\exp\left(-\frac{1}{2\sigma_{p}^{2}}{\left\|p_{i}-p_{j}\right\|}^{2}\right). \end{array}$$

We subsequently obtained 19 characteristics of each object. Each superpixel-SLIC cluster that was characterized was then classified as being either pulmonary parenchyma or a nonpulmonary parenchyma in the first stage of the two-level cascade classification scheme.

**Figure 5:**
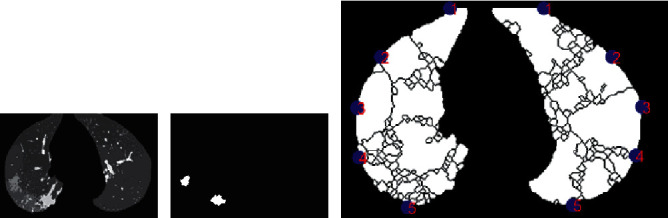
GGO and PI identification. (a) Mean-shift clusters on the identified pulmonary parenchyma zone, (b) results from TRF, and (c) watershed clusters and border regions (blue dots).

The primary contribution of this work is the proposed segmentation and characterization method for the classification of pulmonary parenchyma on CT images (Figure 4(b)). To classify segmented pulmonary parenchyma into CT images, we explored the use of classification approaches that were implemented in Weka (Waikato Environment for Knowledge Analysis) [37, 38]. For the first stage, which is for segmentation and identification of pulmonary parenchyma, we used TRF [39]; the values that were used as parameters in the random forest algorithm are as follows: the number of iterations, 100; the number of trees to be generated, numTrees = 100; the maximum depth of the trees, unlimited maxDepth = 0; and the random number seed to be used, seed = 1. In the second stage in the next section, we identified the GGO and PI from the pulmonary parenchyma region (Figure 5(a)).

Our experimental results (see Section 3) were obtained for this two-level cascade classification scheme.

### 2.4. GGO and PI Identification in Pulmonary Parenchyma

The final proposed part of this second stage was the GGO and PI identification (Figure 5(b)) that was applied to the watershed transform to the mean-shift clusters only in the identified pulmonary parenchyma zone.

Once the pulmonary parenchyma zone on the CT image was identified (Figure 5(a)), segmentation of the watersheds was initialized for this zone (Figure 5(b)). The concept of watersheds [40] in image processing is based on consideration of an image in three-dimensional space, with two spatial coordinates compared to the intensity. The value of the intensity is assumed to be the elevation information. Obtained pixels of watershed lines, which separate neighboring catchment basins and, consequently, separating different characteristic parts of the image, can cluster the oversegmentation that is obtained by the mean shift and focus on finding regions of interest (GGO and PIs).

Because most of the PI usually has denser areas, with a pixel area intensity that is higher than that outside the area, a high gradient and well-delimited image across the infiltrate boundaries is expected. GGO is more textured, and the same high gradient and well-delimited image is expected.

An additional characteristic that is considered on a CT scan that is positive for COVID-19 is GGO, which usually has a bilateral, peripheral, and subpleural distribution, and it is usually predominantly located in the posterior sectors. This is why more weight is given to watershed cluster peripherals for CT screenings that were submitted to our program. Thus, we selected ten specific blue points (Figure 5(c)), five left and five right delimitations of the pulmonary parenchyma borders.

The efficient separation of the true GGO and PI regions from the total watershed regions requires the generation of meaningful features that have very good discriminative ability such as the position Cartesian coordinates [*x, y*] and the first-order statistical average (11), which is the same as (1) that was applied to watershed clusters (*μ*_*w*_).

(11) $$\begin{array}{c} \mu_{w}=\frac{1}{ij}\sum_{i,j}p\left(i,j\right). \end{array}$$

Four second-order statistics texture features, contrast, correlation, energy, and local homogeneity (12)–(15), from the GLCM, which is the matrix *M* as shown below (the measures are calculated for *d* = 0°) and which is the same as (2)–(5), were applied to watershed clusters.

(12) $$\begin{array}{c} cn_{w}=\sum_{i,j}{\left|i-j\right|}^{2}M\left(i,j\right), \end{array}$$

(13) $$\begin{array}{c} cr_{w}=\sum_{i,j}\frac{\left(i-\mu i\right)\left(j-\mu j\right)\vec{p}\left(i,j\right)}{\sigma_{i}\sigma_{j}}, \end{array}$$

(14) $$\begin{array}{c} e_{w}=\sum_{i,j}p\left(i,j\right), \end{array}$$

(15) $$\begin{array}{c} LH_{w}=\sum_{i,j}\frac{p\left(i,j\right)}{1-\left|i-j\right|}. \end{array}$$

We also integrated the entropy features of gradient *X* (16) and gradient Y (17), and based on the entropy, we measured the randomness that can be used to characterize the texture of the gradient *X* and gradient Y in each watershed cluster.

(16) $$\begin{array}{c} S_{GX}=\sum \left(p_{GX}\cdot \;\log\left(p_{GX}\right)\right), \end{array}$$

(17) $$\begin{array}{c} S_{GY}=\sum \left(p_{GY}\cdot \;\log\left(p_{GY}\right)\right). \end{array}$$

For the next ten Euclidean distance features *d* (18), we started from the observation of five left and five right delimitations of the pulmonary parenchyma borders (*br*[*x,y*], (18)). These are regions of interest for finding GGO and PI because they are close to the border regions (10 blue dots, Figure 5(c)). This is the main reason why pulmonary parenchyma is first segmented and identified to obtain five-position Cartesian coordinates [*x, y*] on the left and right sides (10 blue dots, Figure 5(c)). From these ten positions, we obtained the Euclidean distance features *d* (18) with the position Cartesian coordinates of each watershed cluster (*wc*[*x,y*], (18)).

(18) $$\begin{array}{c} d=\sum \sqrt{{\left(br\left[x,y\right]-wc\left[x,y\right]\right)}^{2}}. \end{array}$$

To eliminate false-positive findings, we introduced seven global saliency features GS (19), based on [41]. First, saliency was defined for each watershed cluster as the weighted sum of the cluster's contrast to all other watershed clusters in the segmented image (Figure 5(c)). The weights were set according to the spatial distances with farther regions being assigned smaller weights; salient feature contrast, which characterizes the intensity, the entropy of gradient *X* and gradient Y, and the texture intensity (contrast, correlation, energy, and local homogeneity) provided the seven global saliency features. For a watershed cluster *wc*_*k*_, we computed its saliency value by measuring its feature contrast in relation to all other clusters in the image, where *w* (*wc*_*i*_) is the weight of cluster *i* and *D*_*wc*_(*wc*_*k*_, *wc*_*i*_) is the feature distance metric between the two regions (20).

(19) $$\begin{array}{c} GS\left(wc_{k}\right)=\sum_{wc_{k}\neq wc_{i}}w\left(wc_{i}\right)D_{wc}\left(wc_{k},wc_{i}\right), \end{array}$$

(20) $$\begin{array}{c} D_{wc}=\sum \sqrt{{\left(wc_{k}\left[x,y\right]-wc_{i}\left[x,y\right]\right)}^{2}}. \end{array}$$

We obtained 30 characteristics from each cluster. The main contribution of this work is the proposed identification method for the classification of GGO and PIs (Figure 5(b)). To classify segmented watershed clusters into a normal or abnormal region (GGO and PIs), we have explored the use of the TRF classification approach [39], as described in the last section, and the values used as parameters were numTrees = 100, maxDepth = 0, and seed = 1.

## 3. Experimental Results

### 3.1. Quality Indicators

Several quality indicators have been obtained to quantitatively assess the PP and GGO-PI identification results and the performance of the TRF technique for the first and second stages, respectively.

We divided them into final or external quality indicators, which evaluated the final identification results and are useful for external comparison with other studies, and internal quality indicators, which are useful to evaluate the internal behavior of the proposed classification options (% Training Set – % Test Set).

For the external indicators, we assumed the following: let P be the number of pulmonary parenchyma clusters in the dataset and let TP, FP, and FN be the number of true positives, false positives, and false negatives, respectively. We then defined the following: sensitivity, recall, or true positive rate: TP Rate = TP /(TP + FN); precision or positive predictive value: PPV = TP /(TP + FP); and the F-Measure = (2 × recal1 × precision) /(recall + precision).

Because the proposed algorithm first selects superpixel-SLIC clusters that are then characterized and separated into pulmonary parenchyma and nonpulmonary parenchyma, we can further evaluate the classification performance of the three selected classification schemes via the internal indicators, as follows: let N be the number of nonchest candidates resulting from the application of the proposed method to the complete dataset and let TN be the number of true negatives after classification. We can then define the fall-out or false-positive rate as FP Rate = FP /(FP + TN), and the area under the receiver operating characteristic curve as ROC area.

The above indicators are also applied for ground-truth data for GGO and PI identification.

### 3.2. Quantitative and Qualitative Evaluation of PP and GGO-PI Identification

A medical expert defined a region around the pulmonary parenchyma for a defined ground-truth comparison (see Figure 1(g)–1(l)). The expert also selected and diagnosed the GGO and PI ground-truth comparison (see Figure 1(m)–1(r)). Thus, good and precise segmentation and classification are both desired.

The results of pulmonary parenchyma selection and the feature extraction phases, over the described dataset, are a collection of 43,928 candidate regions as follows: 28,080 nonpulmonary parenchyma (body and background) and 15,848 expected pulmonary parenchyma, which are each characterized by a 19-dimension feature vector for the first stage of the Two-Level Cascade Classification Scheme using TRF. For GGO and PI identification, a collection of 27,340 candidate regions was used as follows: 2,684 normal and 24,692 expected nonnormal (GGO and PIs), which were each characterized by a 30-dimension feature vector for the second stage and which also used TRF.

Table 1 summarizes all of our quantitative results. In the most difficult but more realistic classification experiment, twofold cross validation (i.e., the dataset was divided into two equal parts, one used for training and the other for testing) was used. This finding confirms the reported advantages of TRF over other state-of-the-art classifiers. As expected, as the value of *s* increased in the s-fold cross validation, our results improved until the full set case, in which TRF yielded full precision and recall.

**Figure 6:**
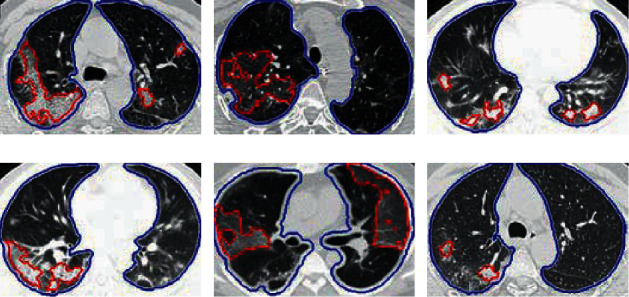
Qualitative identification results of abnormal CT results with COVID-19, including pulmonary parenchyma (blue outline) and GGO and PIs (red outline).

Our qualitative results for identification are shown in Figure 6, with blue superimposed over correctly detected pulmonary parenchyma. These results reveal that the proposed segmentation method can successfully identify pulmonary parenchyma regions. GGO and PIs are indicated with a superimposed red line.

### 3.3. Comparative Discussion

There is a lack of publicly accessible datasets or evaluation scenarios that allow for a fair comparison among methods, and code for the reported methods is unavailable. Thus, we have chosen to present only our results on CT image classification. However, based on the radiographic changes in COVID-19 cases on CT images, many studies have hypothesized that deep-learning artificial intelligence methods could extract the specific graphic characteristics of COVID-19 and provide a clinical diagnosis before pathogenic molecular biological testing, which would save critical time for disease control [9–20, 22–26]. The distinctive characteristics of COVID-19 are the bilateral distribution of irregular shadows with different degrees of radiopacity that are considered to be similar to sperylated or ground glass [2].

One study used a CNN to help with the detection and prediction of COVID-19 [9]. The classifying systems were effective and had an accuracy of 73.1%, specificity of 67%, and sensitivity of 74%. Another study's deep-learning model showed comparable performance to that of an expert radiologist for detecting COVID-19 pneumonia, and the approach provided a classification accuracy of about 95.24% [10]. Another study developed an artificial intelligence-based automated CT image analysis tool for the detection, quantification, and tracking of coronavirus [11]. They achieved classification results for coronavirus versus noncoronavirus with an area under the curve (AUC) of 99.6%.

Another study developed a deep-learning neural network model to extract visual features from volumetric chest CT examinations to detect COVD-19, and the AUC was 96% [12]. A novel learning architecture called Detail-Oriented Capsule Networks (DECAPS) was proposed for the automatic diagnosis of COVID-19 from CT scans. The model achieved 84.3% precision, 91.5% recall, and 96.1% AUC [13]. Another study introduced COVID-Net, a deep CNN design that was tailored to detect COVID-19 cases based on chest X-ray images [14]. For COVID-19 cases, it achieved a good accuracy of 93.3%, a sensitivity of 91.0%, and a high positive predictive value of 98.9%.

Measures of disease severity and a method based on deep learning and deep reinforcement to compute them have been proposed [15]. The Pearson correlation coefficient between the method's prediction and ground truth for positive COVID-19 scans was calculated as 0.92 for the percentage of opacity (*P* < 0.001), 0.97 for the percentage of high opacity (*P* < 0.001), 0.91 for the lung severity score (*P* < 0.001), and 0.9 for the lung high opacity score (*P* < 0.001). Another study compared CNN models to classify CT samples between COVID-19, influenza viral pneumonia, and no infection [16]. We compared that study with one that combined two- and three-dimensional deep-learning models with the latest clinical understanding and achieved an AUC of 0.996 (95% confidence interval (CI): 0.989–1.00) for coronavirus versus noncoronavirus cases of thoracic CT studies. They calculated a sensitivity of 98.2% and a specificity of 92.2%.

An innovative frequency domain algorithm called the FFT-Gabor scheme was proposed, which had an average accuracy of 95.37%, sensitivity of 95.99%, and specificity of 94.76% [17]. Sample-efficient deep-learning methods that can achieve high diagnostic accuracy of COVID-19 from CT scans were developed [18]. The approach achieved an F1 of 0.85 and an AUC of 0.94 in diagnosing COVID-19 from CT scans. Another study classified chest X-ray images with a CNN into two different schemes (first normal and COVID-19; second normal, viral, and COVID-19) [19]. The classification accuracy, precision, sensitivity, and specificity of both schemes were 99.7%, 99.7%, 99.7%, and 99.55% for the first scheme and 97.9%, 97.95%, 97.9%, and 98.8% for the second scheme, respectively.

A deep-learning-based CT diagnosis system (DeepPneumonia) was developed to identify patients with COVID-19 [20]. The model can accurately distinguish COVID-19 patients from others with an excellent AUC of 0.99 and recall (sensitivity) of 0.93. Another study developed a deep-learning-based segmentation system for quantitative COVID-19 infection assessment from chest CT scans [21]. A quantitative evaluation showed high accuracy for automatic delineation of infection regions (dice similarity coefficient = 91.6% ± 10.0%). The iSARF method was proposed, and the experimental results showed that it yielded a sensitivity of 0.907, specificity of 0.833, and accuracy of 0.879 [22].

They proposed a light CNN that was design based on the SqueezeNet model to efficiently distinguish between COVID-19 CT images and other CT images [23]. It had 83.00% accuracy, 85.00% sensitivity, 81.00% specificity, and 81.73% precision and an F1score of 0.8333. Another study proposed a novel COVID-19 lung CT infection segmentation network called Inf-Net [24]. The quantitative results of the detected infection regions included a structure measure of 0.781, enhanced-alignment measure of 0.838, and mean absolute error of 0.082.

A multitask deep-learning model jointly identified COVID-19 patients and segmented COVID-19 lesions from chest CT images [25]. The AUC curve was greater than 93% for the classification. They developed a novel JCS system to make a real-time and explainable COVID-19 diagnosis [26]. It obtained an average sensitivity of 95.0% and a specificity of 93.0% on a classification test set.

The chest CT scan is undoubtedly a tool that helps to obtain a presumptive diagnosis of COVID-19 disease, which provides radiographic images that are suggestive of a multiple focus on pneumonia and COVID-19 disease. The scans are accompanied by the patient's clinical context because the images are characteristic of the disease and can also occur in other types of pneumonia that is caused by microorganisms other than the SARS-CoV-2 virus. However, the chest CT is an imaging tool that provides radiographic information at the thoracic level and is undoubtedly a tool to evaluate the follow-up and prognosis of the disease. RT-PCR is a biomolecular test that is capable of detecting the DNA chain sequence of the SARS-CoV-2 virus. Therefore, it is considered to be the gold standard for a precise diagnosis of COVID-19.

Not many studies have focused on automatically analyzing GGO and PIs on CT scans with COVID-19. The final aim of these studies is to identify GGO and PIs on CT scans with COVID-19, for which GGO and PI identification are considered to be a first and crucial step for timely detection of images that are suggestive of pneumonia secondary to COVID-19. This is an important step because the prognosis and functionality of the patient after recovery from the disease depend on timely treatment.

On the basis of these studies, we can confirm to some extent that our approach is valid based on our TRF classification results for GGO-PI. The GGO-PI identification showed 96% precision and 96% recall on twofold cross validation compared with other similar state-of-the-art studies on CT images. If the analysis is performed on chest CT scans of patients without COVID-19 (50 additional images), better representation with 96.4% precision and 97% recall is obtained.

## 4. Conclusion

Identification of GGO and PI is required for the derivation of diagnostic conclusions and the characterization of the contents in the pulmonary parenchyma area. Automated identification of the GGO and PI in the CT images from these COVID-19 patients is a challenging issue for medical image processing. In this research, we effectively overcame the problem of locating and detecting GGO and PI, and we developed a method to identify pulmonary parenchyma in tomography images. Moreover, we propose a meaningful feature set for the detected clusters, which results in the efficient ability to distinguish between the true abnormal classes (GGO and PIs) using clustering algorithms.

The main advantage of the proposed method is that it can be applied directly to the CT images that were obtained using an X-ray scan, without any observer interference, and it can accurately and in an automated fashion identify first the pulmonary parenchyma and later GGO and PI.

## Figures and Tables

**Table 1:** Quantitative identification results.

Method	% Training Set – % Test Set	External quality indicators			Internal quality indicators	
		TP rate	Precision	F-measure	FP rate	ROC area
PP id (I stage)	2-fcv 0.5–0.5	0.922	0.922	0.922	0.09	0.974
	10-fcv 0.9–0.1	0.975	0.975	0.974	0.186	0.989
GGO-PIs id (II stage)	2-fcv 0.5–0.5	0.968	0.967	0.967	0.237	0.983
	10-fcv 0.9–0.1	0.975	0.975	0.974	0.186	0.989

## Data Availability

Additional materials are available at this link: https://drive.google.com/drive/folders/18qAQqoEFn_ebOY92q-oXNOMZnUEzaufA?usp=sharing. This link contains all the quantitative classification results for download: Folder “Identification images of the lung parenchyma and images of ground-glass opacity and pulmonary infiltrate”. Segmentation of the Pulmonary parenchyma identification images and ground-glass opacity and pulmonary infiltrate images (in bmp format). WEKA PP data features “PP.arff”. 43928 × 20 features in Weka for TRF. Run “PP.arff” for obtained the classification results. GGO-PI data features “GGOPI.arff”. 27340 × 31 features in Weka for TRF. Run “GGOPI.arff” for obtained the classification results.

## References

[1] World Health Organisation (2020). https://www.who.int/news-room/detail/30-01-2020-statement-on-the-second-meeting-of-the-international-health-regulations-(2005)-emergency-committee-regarding-the-outbreak-of-novel-coronavirus-(2019-ncov).

[2] World Health Organisation (2020). https://www.who.int/dg/speeches/detail/who-director-general-s-opening-remarks-at-the-media-briefing-on-covid-19—11-march-2020.

[3] Rubin G. D., Ryerson C. J., Haramati L. B. (2020). The role of chest imaging in patient management during the COVID-19 pandemic. *Chest*.

[4] Shi F., Wang J., Shi J. (2021). Review of artificial intelligence techniques in imaging data acquisition, segmentation, and diagnosis for COVID-19. *IEEE Reviews in Biomedical Engineering*.

[5] Ai T., Yang Z., Hou H. (2020). Correlation of chest CT and RT-PCR testing for coronavirus disease 2019 (COVID-19) in China: a report of 1014 cases. *Radiology*.

[6] Fang Y., Zhang H., Xie J. (2020). Sensitivity of chest CT for COVID-19: comparison to RT-PCR. *Radiology*.

[7] Ng M.-Y., Lee E. Y. P., Yang J. (2020). Imaging profile of the COVID-19 infection: radiologic findings and literature review. *Radiology: Cardiothoracic Imaging*.

[8] Latif S., Usman M., Manzoor S. (2020). Leveraging data science to combat COVID-19: a comprehensive review. *TechRxiv*.

[9] Wang S., Kang B., Ma J. (2020). *A Deep Learning Algorithm Using CT Images to Screen for Corona Virus Disease (COVID-19)*.

[10] Chen J., Wu L., Zhang J. (2020). *Deep Learning-Based Model for Detecting 2019 Novel Coronavirus Pneumonia on High-Resolution Computed Tomography: A Prospective Study*.

[11] Gozes O., Frid-Adar M., Greenspan H. (2020). Rapid AI development cycle for the coronavirus (COVID-19) pandemic: initial results for automated detection & patient monitoring using deep learning CT image analysis. https://arxiv.org/abs/2003.05037.

[12] Li L., Qin L., Xu Z. (2020). Using artificial intelligence to detect COVID-19 and community-acquired pneumonia based on pulmonary CT: evaluation of the diagnostic accuracy. *Radiology*.

[13] Mobiny A., Cicalese P. A., Zare S. (2020).

[14] Wang L., Wong A. (2020). Covid-net: A Tailored Deep Convolutional Neural Network Design for Detection of Covid-19 Cases from Chest Radiography Images. https://arxiv.org/abs/2003.09871.

[15] Chaganti S., Balachandran A., Chabin G. (2020).

[16] Butt C., Gill J., Chun D., Babu B. A. (2020). Retracted article: deep learning system to screen coronavirus disease 2019 pneumonia. *Applied Intelligence*.

[17] Al-Karawi D., Al-Zaidi S., Polus N., Jassim S. (2020). *Machine Learning Analysis of Chest CT Scan Images as a Complementary Digital Test of Coronavirus (COVID-19) Patients*.

[18] He X., Yang X., Zhang S. (2020). *Sample-efficient Deep Learning for COVID-19 Diagnosis Based on CT Scans*.

[19] Chowdhury M. E., Rahman T., Khandakar A. (2020). Can AI help in screening viral and covid-19 pneumonia?. https://arxiv.org/abs/2003.13145.

[20] Song Y., Zheng S., Li L. (2020). *Deep Learning Enables Accurate Diagnosis of Novel Coronavirus (Covid-19) with CT Images*.

[21] Shan F., Gao Y., Wang J. (2020). Lung infection quantification of Covid-19 in CT images with deep learning. https://arxiv.org/ftp/arxiv/papers/2003/2003.04655.pdf.

[22] Shi F., Xia L., Shan F. (2020). Large-scale screening of Covid-19 from community acquired pneumonia using infection size-aware classification. https://arxiv.org/abs/2003.09860.

[23] Polsinelli M., Cinque L., Placidi G. (2020). A Light CNN for Detecting COVID-19 from CT Scans of the Chest. https://arxiv.org/abs/2004.12837.

[24] Fan D.-P., Zhou T., Ji G.-P. (2020). Inf-net: automatic COVID-19 lung infection segmentation from CT images. *IEEE Transactions on Medical Imaging*.

[25] Amyar A., Modzelewski R., Ruan S. (2020). *Multi-task Deep Learning Based CT Imaging Analysis for COVID-19: Classification and Segmentation*.

[26] Wu Y. H., Gao S. H., Mei J. (2004). An Explainable COVID-19 Diagnosis System by Joint Classification and Segmentation. https://arxiv.org/pdf/2004.07054.pdf.

[27] Zhao J., Zhang Y., He X., Xie P. (2003). COVID-CT-dataset: A CT Scan Dataset about COVID-19. https://arxiv.org/abs/2003.13865.

[28] Fukunaga K., Hostetler L. (1975). The estimation of the gradient of a density function, with applications in pattern recognition. *IEEE Transactions on Information Theory*.

[29] Comaniciu D., Meer P. (2002). Mean shift: a robust approach toward feature space analysis. *IEEE Transactions on Pattern Analysis and Machine Intelligence*.

[30] Achanta R., Shaji A., Smith K., Lucchi A., Fua P., Süsstrunk S. (2012). SLIC superpixels compared to state-of-the-art superpixel methods. *IEEE Transactions on Pattern Analysis and Machine Intelligence*.

[31] Haralick R. M. (1979). Statistical and structural approaches to texture. *Proceedings of the IEEE*.

[32] Mariarputham E. J., Stephen A. (2015). Nominated texture based cervical cancer classification. *Computational and Mathematical Methods in Medicine*.

[33] Liu H., Shao Y., Guo D., Zheng Y., Zhao Z., Qiu T. (2014). Cirrhosis classification based on texture classification of random features. *Computational and Mathematical Methods in Medicine*.

[34] Perazzi F., Krähenbühl P., Pritch Y., Hornung A. Saliency filters: contrast based filtering for salient region detection.

[35] Ma Y.-F., Zhang H.-J. Contrast-based image attention analysis by using fuzzy growing.

[36] Adams A., Baek J., Davis M. A. (2010). Fast high-dimensional filtering using the permutohedral lattice. *Computer Graphics Forum*.

[37] Holmes G., Donkin A., Witten I. H., Weka “ A machine learning workbench WEKA: a machine learning workbench.

[38] Garner S. R., Cunningham S. J., Holmes G., Nevill-Manning C. G., Witten I. H. Applying a machine learning workbench: experience with agricultural databases.

[39] Breiman L. (2001). Random forests. *Machine Learning*.

[40] Gonzalez R. C., E Woods R.

[41] Cheng M.-M., Mitra N. J., Huang X., Torr P. H. S., Hu S.-M. (2015). Global contrast based salient region detection. *IEEE Transactions on Pattern Analysis and Machine Intelligence*.

